# Chronic Alcohol Drinking Slows Brain Development in Adolescent and Young Adult Nonhuman Primates

**DOI:** 10.1523/ENEURO.0044-19.2019

**Published:** 2019-04-09

**Authors:** Tatiana A. Shnitko, Zheng Liu, Xiaojie Wang, Kathleen A. Grant, Christopher D. Kroenke

**Affiliations:** 1 Division of Neuroscience, Oregon National Primate Research Center, Beaverton, Oregon 97006; 2 Advanced Imaging Research Center, Oregon Health & Science University, Portland, Oregon 97239; 3 Department of Behavioral Neuroscience, Oregon Health & Science University, Portland, Oregon 97239

**Keywords:** underage drinking, brain growth, magnetic resonance imaging, thalamus, white matter, ethanol, self administration

## Abstract

The transition from adolescence to adulthood is associated with brain remodeling in the final stages of developmental growth. It is also a period when a large proportion of this age group engages in binge alcohol drinking (occasional consumption of four to five drinks leading to intoxication) and heavy alcohol drinking (binge drinking on ≥5 d in a month). Here we report on magnetic resonance imaging of developmental changes in the brain occurring during late adolescence and early adulthood (3.5–7.5 years of age) in a rhesus macaque model of alcohol self-administration. Monkeys were imaged prior to alcohol exposure, and following ∼6 and ∼12 months of daily (22 h/d) access to ethanol and water. The results revealed that the brain volume increases by 1 ml/1.87 years throughout the late adolescence and early adulthood in controls. Heavy alcohol drinking reduced the rate of brain growth by 0.25 ml/year per 1 g/kg daily ethanol. Cortical volume increased throughout this period with no significant effect of alcohol drinking on the cortical growth rate. In subcortical regions, age-dependent increases in the volumes of globus pallidus, thalamus, brainstem, and cerebellum were observed. Heavy drinking attenuated the growth rate of the thalamus. Thus, developmental brain volume changes in the span of late adolescence to young adulthood in macaques is altered by excessive alcohol, an insult that may be linked to the continuation of heavy drinking throughout later adult life.

## Significance Statement

Alcohol abuse during late adolescence and early adulthood is a risk factor for the development of alcohol dependence. This longitudinal study used a macaque model of alcohol self-administration and *in vivo* MRI to quantify the impact of chronic alcohol use on developmental changes occurring within the brain during this period. Chronic alcohol self-intoxication reduced the growth rate of brain, cerebral white matter, and subcortical thalamus. Thus, daily alcohol drinking during the critical transition to adulthood significantly impacts critical areas of sensory motor integration, concomitant with a decrease in cortical white matter, documented in the primate brain neural circuitry implicated in the propagation of alcohol use disorder.

## Introduction

Adolescence is associated with experiencing alcohol binge drinking to extreme intoxication (Patrick and Terry-McElrath, 2017) coincident with the brain maturation processes. *In vivo* magnetic resonance imaging (MRI) studies in humans and other species have confirmed that the brain volume (V_B_) continues to increase throughout childhood and adolescence (Pfefferbaum et al., 1994; Mengler et al., 2014; Scott et al., 2016). The volumetric changes are attributed to synaptic pruning, leading to a reduction of gray matter in the cerebral cortex and an increase in white matter (WM) volume, concurrent in some brain regions, with protracted myelination of late-developing fiber systems (Giedd et al., 1999; Lebel et al., 2008; Sullivan et al., 2011; Yeatman et al., 2014; Levman et al., 2017; Narvacan et al., 2017). In addition, specific age-related growth trajectories have been demonstrated for the medial frontal cortex, thalamus, amygdala, hippocampus, and cerebellum (Barnea-Goraly et al., 2014; Squeglia et al., 2015). Heavy alcohol use in human subjects ranging in age from 9 to 23 years attenuates white matter growth, increases age-related decline in cortical volume, and reduces cortical thickness (Luciana et al., 2013; Squeglia et al., 2015; Pfefferbaum et al., 2016). These studies correspond to imaging experiments in rodents demonstrating that vapor alcohol exposure during adolescence affects cerebral cortical thickness (Vetreno et al., 2017). Chronic alcohol self-administration also attenuated brain growth in a selected line of alcohol-preferring rats (Pfefferbaum et al., 2006). To our knowledge, there are only a couple of reports that have found effects of alcohol on volumetric characteristics of subcortical nuclei. One study found reduced brainstem and caudate volumes in adolescents who engage in heavy alcohol drinking (Squeglia et al., 2014), and another study found a reduction in the volume of the cerebellum, hypothalamus, and hippocampus in alcohol-exposed rodents (Vetreno et al., 2017).

The majority of *in vivo* MRI brain-developmental experiments in humans are cross-sectional due to the resources and time required for a longitudinal design. An exception is the multisite National Consortium on Alcohol and Neurodevelopment (N-CANDA), which is designed to quantify the longitudinal effects of low and heavy alcohol use in children throughout adolescence on the development of cortical regions and the white matter. Importantly, this study includes scanning subjects before any alcohol use (∼9 years of age; Pfefferbaum et al., 2018). The N-CANDA studies included alcohol use assessments, with the caveat that the quantity and frequency of alcohol intake are estimates in human subject studies where high rates of inaccurate self-reported alcohol use might significantly impact the results (Clark et al., 2016; Bertol et al., 2017). The nonhuman primate (NHP) model of alcohol self-administration can control a variety of variables contributing to brain-imaging outcomes, most notably the precise measurements of alcohol intake, diet, daily schedules, and health care (Grant and Bennett, 2003). Therefore, translational MRI research using this model for tracking developmental brain changes might be especially valuable (Zahr and Pfefferbaum, 2017). However, there are relatively few MRI studies that report on overall anatomic change in the maturing NHP brain, especially during late adolescence and early adulthood (Knickmeyer et al., 2010; Scott et al., 2016; Uematsu et al., 2017), much less the effect of alcohol on brain growth parameters. Thus, although reduced cortical gray matter volume was demonstrated in heavy-drinking rhesus macaques, the study was underpowered to specifically address changes in brain growth when chronic ethanol drinking begins in late adolescence (Kroenke et al., 2014).

This gap in our knowledge was addressed in the present study, which measured volumetric trajectories occurring in the rhesus macaque brain during the period of late adolescence to early adulthood and how these changes are affected by chronic alcohol self-administration. We tested the hypothesis that chronic alcohol self-administration dose-dependently decreases whole brain, cortical, and white matter volumes in the macaque. We also predicted that subcortical structures undergoing growth during this age range would also be vulnerable to alcohol-dependent effects on volume in the heavy drinker (HDs).

## Materials and Methods

### Animals

Male (*n* = 58) and female (*n* = 13) rhesus monkeys (*Macaca mulatta*; *N* = 71; cohorts 4, 5, 6a, 6b, 7a, 7b, 10, and 14; for details, see www.matrr.com) were obtained from the Oregon National Primate Research Center (ONPRC) breeding colony. All monkeys except cohort 4 were enrolled in the study at 3.5–5.5 years of age and 3–6 kg in body weight. All animals were housed indoors in rooms with controlled temperature (20–22°C), humidity (65%), and an 11 h light cycle with lights on at 7:00 A.M. Each subject was housed in a metal cage (0.8 × 0.8 × 0.9 m) and fed a diet of nutritionally complete 1 g of banana-flavored pellets (TestDiet) and fresh fruit. Food and fluid availability was dependent on experimental phase, as described below. All monkeys were weighed weekly, and ethanol intake based on body weight (in grams per kilogram) was calculated from the contemporary weights. All procedures were conducted according to the *Guide for the Care and Use of Laboratory Animals* (National Research Council, 2011) and approved by the Oregon National Primate Research Center Animal Care and Use Committee.

### Alcohol self-administration

#### Equipment

The housing cages were equipped with operant panels as previously described (Vivian et al., 2001; Shnitko et al., 2017). Each panel incorporated a centrally located dowel and two drinking spouts, with a food receptacle located below one of the spouts. The receptacle was connected to a 1 g pellet dispenser (Med Associates). Below the receptacle, there was a recessed well that could detect the insertion of a finger via the breaking of an infrared beam. Each drinking spout was connected via tubing to a plastic bottle placed on a digital scale (Adventurer, Ohaus) located outside the cage. The bottle contained either filtered tap water or 4% ethanol (w/v, diluted in water), refilled with fresh solutions daily. All programming used a National Instruments interface and LabView software (LabView 2011, Service Pack 1, National Instruments). Thus, the operant panels were used for the ethanol and water self-administration and food delivery. To initiate food or fluid delivery, the dowel had to be pulled (closing an electrical circuit) and held (for details, see Grant et al., 2008).

#### Procedure to induce alcohol self-administration

Alcohol self-administration began after a baseline MRI assessment was completed. As previously described, a schedule-induced polydipsia procedure was used to induce monkeys to drink 4% (w/v) ethanol (Vivian et al., 2001; Grant et al., 2008). The induction sessions were given daily over a period of ∼4 months (∼120 sessions, 7 d/week). During each session, 1 g of banana-flavored pellets were delivered at a fixed time interval of 300 s until a required volume of either water or 4% (w/v) ethanol was consumed. Initially, monkeys were induced to drink water for 30 sessions (1 session/d), and then water was replaced with 4% ethanol (for monkeys assigned to ethanol experimental group). Monkeys were required to consume the ethanol solution at a volume sufficient to obtain 0.5 g/kg/d (30 d), 1.0 g/kg/d (30 d), and 1.5 g/kg/d (30 d) ethanol. Control (CTR) animals were required to drink water at the volumes corresponding to the ethanol doses. When an animal consumed the required volume, the ethanol spout became inoperative, but water was available through the other spout, and the remaining daily ration of food became available 2 h later (Grant et al., 2008).

#### Open access to alcohol self-administration

**Figure 1.**
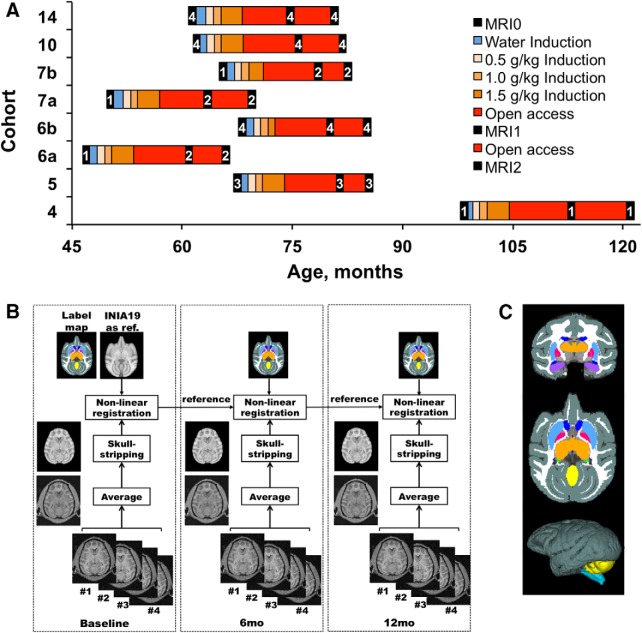
***A–C***, Schematic representations of experimental timeline (***A***) and MRI data processing (***B***, ***C***). ***A***, The timelines of the experiment are shown for each of eight cohorts of subjects admitted to and completed the study at different periods from 2008 to 2017. As indicated in the legend, the black squares represent an MRI sessions in the timeline of the experiment, and the numbers within them indicate an MRI protocol used at the time. ***B***, The MR image processing is described in detail in the text (see Materials and Methods). ***C***, Representation of the brain map of the monkey used for segmentation of ROIs in individual subjects (top, coronal; middle, axial; bottom, 3D image showing cortical surface, the cerebellum, and brainstem). The ROIs were labeled in INIA19 template brain.

When induction to ethanol drinking was completed, all animals were allowed daily “open access” to water and ethanol (4% w/v, or water for control subjects) for 22 h/d, 7 d/week (Grant et al., 2008). During the open access period, the daily food ration (banana-flavored pellets) was divided equally into three meals; the first meal was available at the beginning of each daily session, and subsequent meals were available at 2 h intervals. The pellets were available under a fixed ratio 1 schedule. Initially, animals were allowed ∼6 months of concurrent access to water and 4% (w/v) ethanol, for a total of 201 ± 19 sessions (*N* = 61, including only adolescent cohorts; i.e., cohort 4 excluded). Subsequently, an additional 171 ± 16 self-administration sessions were allowed (∼12 months of open access). The study timeline for each cohort is shown in Figure 1*A*.

#### Blood ethanol concentration

Before MRI and ethanol self-administration sessions, all animals were trained to comply with veinipuncture procedure without the use of anesthesia. Initially, subjects were trained to present their leg through an opening in the front wall of the housing cage. They then were trained to comply with blood sampling to be reinforced with raisins or trail mix. Blood samples were collected from the saphenous vein 30, 60, and 90 min after the start of the induction sessions and 7 h after the start of the 22 h drinking sessions in 4–5 d intervals. The blood samples were collected throughout the 3 months of ethanol induction and period of open access to ethanol. Ethanol content was assessed by gas chromatography (5890 Series II, Hewlett-Packard).

### MRI data acquisition and processing

Before an imaging session, each animal was anesthetized with ketamine (15 g/kg, i.m.) in the housing environment. The animal was transferred to the ONPRC MRI Core Facility, intubated and maintained anesthetized with 1–1.5% isoflurane throughout imaging procedures (≤2 h). The images were acquired using a 3 T Siemens Magnetom MRI system. For all subjects in this study, the MRI protocol included an acquisition of T_1_-weighted magnetization-prepared rapid acquisition gradient echo (MP-RAGE) with one of the four imaging protocols described in Table 1. The imaging data were acquired at baseline (before alcohol self-administration, MRI_1_), after 6 months of self-administration (MRI_2_), and after 12 months of self-administration (MRI_3_). Within each cohort, image acquisition settings were kept constant, except for cohorts 6a, 7a, and 7b, in which the MRI system was upgraded from a Trio to a Tim Trio between the MRI_1_ and MRI_2_ time points, and different MRI protocols were used for data acquisition in the cohorts. Between cohorts, experiments took advantage of upgrades in radiofrequency coil or scanner capabilities as they became available.

**Table 1:** Protocols of MR image acquisition

Protocol	MRI system	RF coil	TR (ms)	TE (ms)	TI (ms)	Voxel size (mm)	Flip angle
1	Trio	Quadrature knee	2500	4.38	1100	0.5 × 0.5 × 0.5	12
2	TimTrio	Quadrature knee (Tim)	2500	3.86	1100	0.5 × 0.5 × 0.5	12
3	Trio	8-Channel knee array	2500	4.38	1100	0.5 × 0.5 × 0.5	12
4	TrioTim	15-Channel knee array	2500	3.86	1100	0.5 × 0.5 × 0.5	12

RF, Radiofrequency. As indicated in the table, the ONPRC MRI system was upgraded throughout the study and different RF coils were used to enhance the signal-to-noise ratio. The parameters of MR image acquisition were adjusted accordingly.

### MRI data processing

Figure 1*B* shows a schematic representation of the processing for volumetric analysis within a NHP brain. At the beginning, all T1-weighted images collected at a given time point were averaged within a subject after motion correction and intensity bias correction. The motion correction was implemented by the rigid-body registration with “antsRegistrationSyN.sh,” which is an Advanced Normalization Tools (ANTS) standard function (Avants et al., 2008). The intensity bias field of each T1-weighted image was corrected using a B-spline approximation routine and a hierarchical optimization scheme implemented by “N4BiasFieldCorrection” in ANTS (Tustison et al., 2010). Next, a registration-based skull-stripping procedure was applied to the averaged MP-RAGE images successively from MRI_1_ to MRI_3_. At the beginning, the INIA19 template, which includes a T1-weighted head image, brain mask, and NeuroMap labels, was used a reference (Rohlfing et al., 2012). All corrected and averaged T1-weighted images in MRI_1_ were nonlinearly registered to the INIA19 head image using “antsRegistrationSyN.sh.” With the resulting transformation parameters, the INIA19 brain mask was then reversely aligned to each subject image to generate the brain mask using a nearest neighbor interpolation method. For MRI_2_ and MRI_3_, skull stripping was performed with the same method but updated references (MRI_2_ with MRI_1_ as reference, and MRI_3_ with MRI_2_ as reference). Based on the registration results, the label map of the INIA19 template was transformed to the space of each MRI_1_ image. With the same method, the label maps of brain images in MRI_2_ and MRI_3_ were generated based on the resulting transformation parameters. Subsequent to the analysis of brain volume changes, a secondary analysis was performed to determine whether individual brain regions are differentially susceptible to age or ethanol exposure. Accordingly, we explored effect of ethanol on age-related changes on a finer scale by parcellating the brain volume into the following 10 well resolved regions of interest (ROIs): WM, cortex, putamen, caudate nuclei, globus pallidus, thalamus, amygdala, hippocampus, brainstem, and cerebellum, illustrated in Figure 1*C*. The ROI boundaries were defined before the statistical analysis of the determined volumes.

### Statistical data analysis

Imaging data for all subjects collected longitudinally across three time points were analyzed using a linear mixed model (LMM). A series of LMMs were used in this study to estimate the effects of age and chronic ethanol drinking on volumetric characteristics of the primate brain where *Y_i_*_,_*_j_* is V_B_, white matter volume (V_WM_) or volume of an ROI, as follows:

LMM 1: *Y_i_*_,_*_j_* ∼ β_0_ + β_1_age*_i_*_,_*_j_* + *b_j_*subject *+* ε*_i_*_,_*_j_*

LMM 2: *Y_i_*_,_*_j_* ∼ β_0_ + β_1_age*_i_*_,_*_j_*+ β_2_intake_i,j_ + β_3_age*_i_*_,_*_j_* × intake*_i_*_,_*_j_* + *b_j_*subject *+* ε*_i_*_,_*_j_*

LMM 3.1: *Y_i_*_,_*_j_* ∼ β_0_ + β_1_age*_i_*_,_*_j_* + β_2_group*_i_*_,_*_j_* + β_3_age*_i_*_,_*_j_* × group*_i,j_* + b_j_subject *+* ε*_i,j_*

LMM 3.2 *Y_i_*_,_*_j_* ∼ β_0_ + β_1_age*_i_*_,_*_j_* + β_2_group*_i,j_* + β_3_sex*_i,j_* + β_4_age*_i,j_* × group*_i,j_* + β_5_age*_i,j_* × sex*_i,j_* + β_6_group*_i,j_* × sex*_i,j_* + β_7_age_i,j_ × group*_i,j_* × sex*_i,j_* + *b_j_*subject *+* ε*_i,j_*

First, we estimated age-related change in the V_B_ of CTR animals using LMM 1, which incorporates a fixed effect of age (β_1_) and a random intercept (*b_j_*). Next, we explored whether the age effect on *Y_i_*_,_*_j_* depends on ethanol intake using LMM 2 with fixed effects of age and average ethanol intake (β_2_), as well as an age-by-intake interaction (β_3_), and a random intercept.

Subjects were categorized as low, binge, heavy, and very heavy drinkers based on the ethanol intake parameters of each individual during the open access period, as described in previous studies (Baker et al., 2014). Briefly, an animal was considered to be a low drinker if its average ethanol intake per day was <2 g/kg; an animal was considered a binge drinker if similar ethanol intake per day (<2 g/kg) resulted in at least a single blood ethanol concentration (BEC) value >80 mg%. The demarcation for heavy and very heavy drinkers was an average ethanol intake >3 g/kg during 30% of total given 22 h sessions, and >4 g/kg during 20% of total given 22 h sessions, respectively. The low and binge drinkers were collapsed into a group of non-HDs (NHDs; *n* = 19), and heavy and very heavy drinkers were grouped as HDs (*n* = 26). This parallels previous work in humans (Squeglia et al., 2015) and in nonhuman primates (Cervera-Juanes et al., 2017; Shnitko et al., 2019). The third linear mixed model (LMM 3.1) used the categorical variable (group) to determine how heavy and nonheavy alcohol drinking affects age-related changes in volume of the brain, white matter, and gray matter. If a significant effect of group or an age-by-group interaction was found, then the age-dependent changes were compared with the CTR group as a reference. Finally, we used LMM 3.2 to explore whether sex has a significant effect on age-related changes in the volume of the brain and interacts with effects of heavy and nonheavy alcohol drinking. All statistical analysis was performed in SPSS Statistics, version 24 (IBM). All significant effects of main factors and interactions were confirmed with an *F* test within each model indicated above. For the analysis of age-dependent change in the total brain volume, a significance threshold of *p* < 0.05 was considered to be significant. For the analysis of age-dependent change in the volume of the 10 ROIs, a significance threshold of *p* < 0.005 (adjusting for 10 ROIs) was considered significant. The significant fixed effects and interactions found in linear mixed model analyses were followed by *post hoc t* tests in which *p* values were adjusted for the number of comparisons, and differences of *p* < 0.05 were considered to be significant.

## Results

### Brain growth in late adolescent/young adult rhesus monkeys

**Figure 2.**
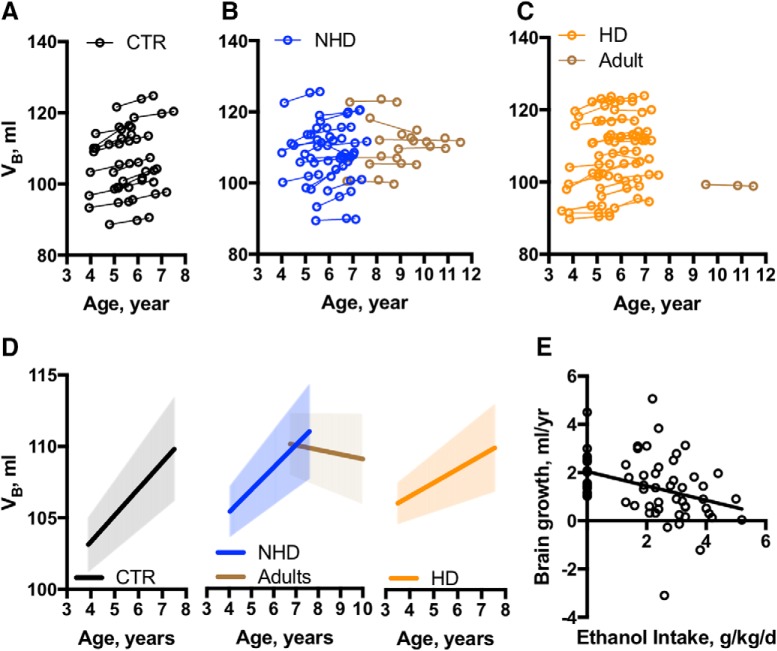
Age-dependent brain growth in the nonhuman primates. ***A–C***, Plots of individual brain volume changes across age in CTR (*n* = 16), NHD (*n* = 19), and HD (*n* = 26) NHPs. The V_B_ of each subject was measured three times, as depicted by empty circles. An individual regression line is drawn across the three V_B_ values for each subject. ***D***, Average V_B_-by-age linear regression estimated for CTRs, NHDs, and HDs with 95% confidence interval depicted by the shaded area around the line. ***E***, Correlation between age-dependent brain growth (β*_i_*) and individual daily ethanol intake averaged across 12 months of drinking. The β*_i_* value is based on the slope of the individual regression line, as shown in ***A–C***. The ethanol intake is estimated based on daily consumption of 4% (w/v) ethanol solution averaged across ∼372 drinking sessions with the exception of the induction period (*r_s_* = 0-0.41, *p* < 0.01).

Subjects were initially studied (MRI_1_) at ages ranging from 3.9 to 5.9 years and reached MRI_3_ at ages ranging from 5.6 to 7.5 years (Fig. 1*A*). First, we wanted to establish whether brain continues to grow in the NHPs over the age range of 3.9–7.5 years (Fig. 2*A*). The average brain volume across all CTR animals was 104.6 ± 9.4 ml at the MRI_1_, 105.9 ± 11.4 ml at the MRI_2_, and 107.1 ± 8.5 ml at the MRI_3_. Analysis of V_B_ using LMM 1 revealed a significant effect of age on V_B_ [β = 1.87, SE = 0.17, *p* < 0.00001; 95% confidence interval (CI), 1.5–2.1]; the V_B_ of CTR animals increases by 1 ml/1.87 years.

### Voluntary ethanol intake during open access

After the induction period, 55 of the 71 animals were allowed to voluntarily self-administer ethanol during a 12 month period (Fig. 1*A*, open access). The average number of open access drinking days was 179 ± 13 before MRI_2_, and 164 ± 11 between MRI_2_ and MRI_3_. The average ethanol intake of NHD animals was 1.8 ± 0.5 and 2.1 ± 0.4 g/kg prior to MRI_2_ and between MRI_2_ and MRI_3_, respectively (Table 2). Low ethanol intake was reflected in the low levels of BEC collected 7 h after the initiation of drinking sessions. The group average BECs of NHD animals was 30 ± 7.6 mg% (individual averages ranged from 3 to 73 mg%), and 37 ± 7.2 mg% (individual averages ranged from 3 to 100 mg%) before and after MRI_2_, respectively. The average ethanol intake of HD animals was 3.0 ± 0.3 and 3.4 ± 0.4 g/kg during the same periods. Higher ethanol intake in the HD group resulted in relatively high group average BECs of 75 ± 19 mg% (individual averages ranged from 21 to 159 mg%) averaged across drinking sessions before MRI_2_ and 105 ± 24.2 mg% (individual averages ranged from 6 to 196 mg%) averaged across sessions between MRI_2_ and MRI_3_.

**Table 2:** Data sample characteristics

	Age (SD), year			Ethanol Intake (SE), g/kg/d		
MRI	CTR	NHD	HD	CTR	NHD	HD
1	4.7 (0.6)	5.1 (0.6)	4.8 (0.8)	0.0	0.0	0.0
2	5.8 (0.6)	6.2 (0.6)	5.9 (0.7)	0.0	1.8 (0.5)	3.3 (0.8)
3	6.3 (0.6)	6.6 (0.6)	6.4 (0.7)	0.0	2.1 (0.4)	3.6 (0.8)

One-way ANOVA revealed no significant difference in age at the beginning of the study (MRI 1) among three groups of subjects (*F*_(2,60)_ = 1.3, *p* = 0.28). Repeated-measures ANOVA revealed significant effects of MRI (2 vs 3: *F*_(1,43)_ = 11.3, *p* < 0.01) and group (NHD vs HD: *F*_(1,43)_ = 67.9, *p* < 0.0001) on ethanol intake with no significant interaction (*F*_(1,43)_ = 0.06, *p* = 0.8).

### Heavy drinking attenuates growth in the late adolescent/young adult rhesus brain in an age-dependent manner

To explore whether chronic ethanol self-administration affects brain growth in nonhuman primates, we used LMM2 with age at MRI sessions 1-3, average ethanol intake, and the potential interaction between them as predictors of V_B_. This analysis identified that age (β = 1.9, SE = 0.2, *p* < 0.00001; 95% CI, 1.5–2.3) and ethanol intake (β = 1.2, SE = 0.5, *p* < 0.05; 95% CI, 0.24–2.2) significantly predicted V_B_, and these effects were interdependent, as demonstrated by a statistically significant age-by-intake interaction (β = −0.25, SE = 0.08, *p* < 0.01; 95% CI, −0.4 to −0.09). Thus, brain growth occurred in all NHP subjects studied over this range; however, it was attenuated by a factor of 0.25 ml/year per daily gram per kilogram ethanol. Figure 2*E* shows a correlation between individual brain growth (β*_i_*) and average ethanol intake of the subject across 12 months of open access (Spearman correlation, *r_s_* = −0.41, *p* < 0.01).

An additional analysis was performed in which ethanol-drinking monkeys were categorized as NHDs and HDs. Drinking group (CTR animals, NHDs, and HDs) was used as a fixed factor in LMM 3.1 to compare age-related brain growth between subjects with different drinking status. Figure 2, *B* and *C*, shows brain growth trajectories obtained based on data collected longitudinally during the three MRI sessions in NHD and HD monkeys. The LMM 3.1. analysis identified that age significantly predicts V_B_ (*F*_(1,123)_ = 141.9, *p* < 0.0001); however, the age-related V_B_ was dependent on drinking group (age × group interaction: *F*_(2,123)_ = 5.5, *p* < 0.01). Multiple comparisons between groups (Fig. 2*D*) show that the age-dependent increase of V_B_ in HDs was significantly attenuated when compared with CTR animals (β´ = −0.9, SE = 0.3, adjusted *p* = 0.004; 95% CI, −1.5 to −0.3), while a similar rate of increase of V_B_ was observed in CTRs and NHDs (β´ = −0.3, SE = 0.64, *p* = 0.32; 95% CI, −0.9 to −0.3). Importantly, the age-dependent changes in the V_B_ significantly differ between two groups of NHD animals (late adolescent and adult) as depicted in Figure 2*D*. A linear mixed-model analysis revealed a significant effect of the groups (*F*_(1,71)_ = 8.6, *p* < 0.01) on the age-related changes in V_B_. The brain continues to grow in younger NHD animals throughout the period from 4 to 7.5 years of age. In adult subjects, the growth stopped and V_B_ declined by 0.27 ml/year. This differed from the adolescent NHD by 1.8 ml/year (β´ = 1.8, SE = 0.4, *p* = 0.0001; 95% CI, −0.9 to 2.6). Importantly, as indicated in Table 1, multiple hardware configurations were used as a result of MRI system upgrades over the course of this study. An analysis of the potential effect of MRI protocol on the results reported here was conducted using a mixed-model analysis. This revealed no significant effect of data acquisition parameters (effect of MRI protocol: *F*_(3,73)_ = 1.4, *p* = 0.24; effect of age: *F*_(1,123)_ = 68.7, *p* < 0.0001; effect of group: *F*_(2,100)_ = 1.4, *p* = 0.25; age × group interaction: *F*_(1,123)_ = 5.5, *p* < 0.01).

Last, we used the small subset of female subjects in this study (*n* = 13) to examine the possibility that the effects of alcohol exposure on brain growth was sex dependent. The analysis using LMM 3.2 revealed a significant effect of sex on V_B_ (*F*_(1,122)_ = 91.4, *p* < 0.0001), but no significant interactions between sex and age (*F*_(1,122)_ = 0.4, *p* = 0.5) and between sex and group (*F*_(2,120)_ = 1, *p* = 0.35), or a sex × age × group interaction (*F*_(2,122)_ = 1.7, *p* = 0.2). These findings correspond to previous reports demonstrating sex-dependent differences in the brain volume of humans and nonhuman primates (Leonard et al., 2008).

### The white matter and the thalamus are the most susceptible to chronic drinking during late adolescence

Previous studies have demonstrated varying age-dependent changes in the volume of different brain structures (Sowell et al., 2004; Sullivan et al., 2011; Dennison et al., 2013; Barnea-Goraly et al., 2014; Yeatman et al., 2014; Bernard et al., 2015) and have also shown that heavy alcohol drinking specifically attenuates white matter growth and decreases the volume of other brain structures such as the cortex, brainstem, caudate, hippocampus, and cerebellum (Luciana et al., 2013; Kroenke et al., 2014; Vetreno et al., 2017). To determine whether ethanol differentially affects the development of different brain regions, the brain was parcellated into 10 ROIs, and ethanol effects were examined within each of them separately, using LMM 3.1. The results of tests of fixed effects are presented in Table 3. According to our criteria, there were no significant age-related changes in the volume of caudate nucleus, hippocampus, putamen, cortex, and amygdala, as indicated by a lack of significant effects of age and group × age interaction (*p* < 0.005). However, age-dependent changes in volumes were identified for the cerebellum, brainstem, globus pallidus, thalamus, and white matter. The cerebellum, brainstem, and globus pallidus exhibit a statistically significant effect of age, but no age × group interaction. As shown in Table 4, the rates of growth in these structures were estimated to be 0.14, 0.012, and 0.16 ml/year in the cerebellum, globus pallidus, and brainstem, respectively. Thus, the brainstem volume undergoes the highest change (5% per year from the baseline level) during the age period from 3.9 to 7.9 years compared with two other structures: the globus pallidus (3.2%/year) and the cerebellum (1.6%/year).

**Table 3:** Tests of fixed effects in LMM 3.1

	Groupa		Agea		Interaction	
ROIa	*F*	*P*	*F*	*p*	*F*	*p*
WM	4.4	<0.05	405.2	<0.00001	9.6	<0.00001
Thalamus	3.7	<0.05	108.8	<0.00001	8.2	<0.00001
Globus pallidus	1.6	0.2	148.4	<0.00001	3.7	<0.05
Brainstem	1.1	0.3	700.7	<0.00001	3.9	<0.05
Cerebellum	1.6	0.2	120.5	<0.00001	1	0.4
Amygdala	1.6	0.2	12.7	<0.01	3.2	<0.05
Cortex	1.9	0.2	11.4	<0.01	2.2	0.1
Putamen	0.8	0.4	10.8	<0.01	1.9	0.2
Caudate	2.3	0.1	0.7	0.4	0.7	0.5
Hippocampus	1.8	0.2	0.2	0.6	2.3	0.1

The table includes the results of the test for fixed effects used in the LMM3.1: age, group, and age × group interaction. Note that the volume of a single ROI was used as a dependent variable in LMM3.1.

*^a^*The degrees of freedom for the numerator and denominator are 1 and 123 (age), 2 and 96 (group), and 2 and 123 (interaction), respectively. Note that only *p* values <0.005 were considered significant (adjustment for 10 ROIs included in the analysis).

**Table 4:** Estimates of fixed effects in LMM 1

ROI	β	SE	*p*	95% CI
Cerebellum	0.14	0.01	<0.00001	0.12–0.17
Globus pallidus	0.012	0.001	<0.00001	0.009–0.1
Brainstem	0.16	0.006	<0.00001	0.15–0.018

The table includes the parameters of estimates of fixed effects obtained in the LMM1 with random intercept and fixed effect of age on V of listed ROIs. The rate of volume change is expressed as milliliters per year.

**Figure 3.**
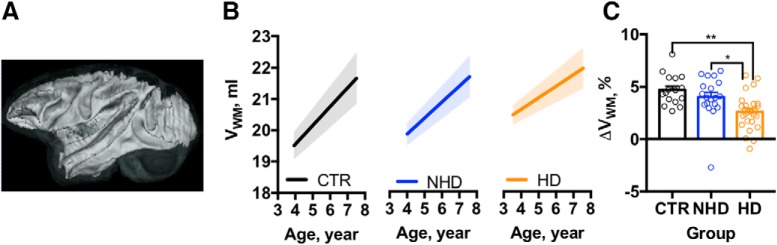
Heavy ethanol intake reduces the rate of the white matter growth in NHP brain. ***A***, 3D representation of the cortical white matter in the brain. ***B***, The estimated rate of white matter growth in the control, non-heavy-drinking, and heavy-drinking NHPs. The shadows above and below the regression lines depict the 95% confidence interval. ***C***, The effect of chronic ethanol use on the average change in white matter volume occurred in three groups of subjects from baseline until the end of ethanol/water self-administration. The dots represent change in the volume measured in individual monkeys. Asterisks show the results of Bonferroni *post hoc* test, where *p* values adjusted for the multiple comparisons were **p < 0.01 and *p < 0.05.

As shown in the Table 3, the white matter and thalamus also exhibit significant age-related changes in their volumes; however, the volumetric changes were ethanol intake dependent. Table 5 demonstrates the estimated effects of age on volume for these ROIs within CTR, NHD, and HD groups. The analysis with LMM 3.1. revealed a striking effect of heavy drinking on the age-related growth in the white matter and the thalamus. The whole-brain white matter continued to increase during the period from 3.9 to 7.9 years in all subjects (Fig. 3*B*).

**Table 5:** Estimates of fixed effects in LMM 3

CTR					NHD				HD			
ROI	β	SE	*p*	95% CI	β´	SE	Adjusted *p*	95% CI	β´	SE	Adjusted *p*	95% CI
WM	0.6	0.050	<0.00001	0.5-0.7	−0.07	0.06	0.44	−0.2 to 0.04	−0.25	0.06	<0.00001	−0.4 to −0.13
Thalamus	0.06	0.007	<0.00001	0.04-0.07	−0.02	0.01	<0.01	−0.4 to −0.01	−0.03	0.01	<0.00001	−0.05 to −0.02

The table includes the results obtained with the LMM 3.1 with random intercept and fixed effect of age, group, and their interaction on V of the ROIs. β´ = βNHD or HD-βCTR. The rate of volume change is expressed as milliliters per year. Average estimated age-dependent growth trajectories obtained in the NHD and HD groups were compared with those of the CTR group, with *t* test and *p* values adjusted accordingly.

In the CTR group, the estimated rate of V_WM_ growth was 0.6 ml/year, and it was slightly lower in NHDs by 0.07 ml/year compared with CTR animals. However, the rate of growth was robustly attenuated in HDs compared with CTR animals by 0.25 ml/year. To confirm that there were no group differences in the V_WM_ at baseline (MRI_1_) before ethanol self-administration, we performed ANOVA. The analysis revealed no significant differences among three groups of monkeys (*F*_(2,58)_ = 1.1, *p* = 0.35), where the average V_WM_ at MRI_1_ was 19.9 ± 2 ml in CTR animals, 20.4 ± 1.7 ml in NHDs, and 20.9 ± 2.2 ml in HDs. Figure 3*C* shows the change in the volume of WM that occurred between baseline (MRI_1_) and the end of alcohol or water (control) self-administration (an ∼12 month period). In the CTR monkeys, V_WM_ increased by 4.7 ± 0.4%. Similar to CTR animals, the 4.0 ± 0.5% increase in the V_WM_ was observed in NHDs. In HDs, the increase in the V_WM_ was significantly smaller (2.6 ± 0.3%) compared with CTR. One-way ANOVA revealed a significant effect of group (*F*_(2,58)_ = 7.8, *p* < 0.001; Fig. 3*C*, results of *post hoc* analysis).

**Figure 4.**
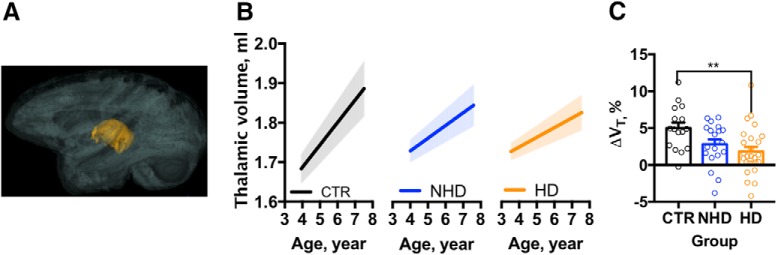
Ethanol drinking attenuates thalamic growth in adolescent/early adult NHP. ***A***, 3D representation of the thalamus in the brain. ***B***, The estimated rate of the thalamic growth in the control, non-heavy-drinking, and heavy-drinking NHPs. The shadows above and below the regression lines depict 95% confidence interval. ***C***, The effect of chronic ethanol use on the average change of thalamic volume occurred in three groups of subjects from baseline until the end of ethanol/water self-administration. The dots represent change in the volume measured in individual monkeys. Asterisks show the results of a Bonferroni *post hoc* test, where *p* values adjusted for the multiple comparisons were **p < 0.01.

Thalamic growth also occurred in the NHP brain over this period (Fig. 4*B*). In the CTR group, the estimated rate of growth was 0.06 ml/year. It was significantly decreased in both types of alcohol drinkers, NHDs (by 0.02 ml/year) and HDs (0.03 ml/year). We confirmed that there were no group differences in the thalamic volume (V_T_) at baseline (MRI_1_) before ethanol self-administration using ANOVA. The analysis revealed no significant differences among the three groups of monkeys (*F*_(2,58)_ = 0.3, *p* = 0.73), where the average V_T_ at MRI_1_ was 1.7 ± 0.2 ml in CTR animals, 1.7 ± 0.15 ml in NHDs, and 1.7 ± 0.13 ml in HDs. The V_T_ increased by 5 ± 0.7% between baseline (MRI_1_) and the end of self-administration in CTR animals and by 2.8 ± 0.6% in NHDs. In HDs, the 1.8 ± 0.6% increase in the V_T_ was significantly smaller, as revealed as a significant effect of group in one-way ANOVA (*F*_(2,58)_ = 5.5, *p* < 0.01; Fig. 4*C*, results of *post hoc* analysis).

## Discussion

Rhesus macaques have been widely used as a translational model for investigating the neural substrates of human behavior and, particularly, alcohol use and abuse (Grant and Bennett, 2003; Wright and Taffe, 2014; Chandler et al., 2017; Shnitko et al., 2019; Thomas and Czoty, 2019). The present longitudinal study was focused on the measurement of brain changes that occur during late adolescence and early adulthood in rhesus monkeys (3.5–7.5 years of age) because this stage of development confers maximum risk for heavy drinking in humans and macaques (Helms et al., 2014; Substance Abuse and Mental Health Services Administration, 2017). We quantified changes in the volume of brain structures in macaques over this period of life and characterized the effects of chronic alcohol use on these changes. First, we demonstrated that the brain of the macaque continues to grow well into young adulthood (at least until 7.5 years of age), as previous studies have not measured brain volume increases beyond adolescence in the macaque (until 5 years of age; Malkova et al., 2006; Bakken et al., 2016; Scott et al., 2016). Second, the reduced rate of brain growth due to heavy ethanol drinking could be quantified with this analysis, and was found to be 0.25 ml/year per gram per kilogram daily ethanol intake. Finally, these results extend the chronic effects of heavy alcohol intake from brain volume reductions in adult rhesus macaques to significant impact on brain growth at an age range associated with a high risk of establishing a pattern of unhealthy alcohol consumption (Kroenke et al., 2014).

Although the rate of brain volumetric growth decreases during development (Bakken et al., 2016), the present data clearly show that brain continues to grow throughout late adolescence and early adulthood (Fig. 2*A*). A diverse set of neurodevelopmental processes contribute to this volume change. For example, the cortical volumes of gray and white matter undergo nonlinear changes across the human life span, with gray matter volume decreasing during late adolescence and early adulthood, while white matter volume continues to increase beyond adolescence and early adulthood (Sowell et al., 2003, 2004). A regressive neuronal process contributing to the gray matter volumetric changes is the synaptic pruning that begins in childhood and continues in adulthood (Sowell et al., 2001; Chung et al., 2017). In rhesus macaques, the initiation of synaptic pruning occurs between 1 and 3 years of age and accelerates during puberty (Eckenhoff and Rakic, 1991; Zecevic and Rakic, 1991). At the same time, myelination of many white matter fascicles is quite protracted over adolescent development in humans and NHPs (Sowell et al., 2001; Miller et al., 2012), which contributes to the increase in white matter volume throughout adolescence. As stated above, the present data are the first to document normal developmental changes in the brain volume beyond 5 years of age in rhesus macaques.

These neurodevelopmental underpinnings are thought to form the biological bases of robust behavioral and cognitive changes that occur concurrently and are accompanied by susceptibility to maladaptive behaviors in adolescents. Importantly, the age at onset of alcohol drinking is considered as one of the significant predictors of heavy alcohol drinking in adulthood (Poikolainen et al., 2001; Englund et al., 2008; Morean et al., 2012; but see Maimaris and McCambridge, 2014). Furthermore, recent N-CANDA experiments found robust effects of chronic alcohol abuse on brain volumes in human adolescents (Squeglia et al., 2015; Pfefferbaum et al., 2016, 2018). Similar to human subjects, macaque individuals that begin drinking to intoxication as late adolescents/young adults have a greater risk for future heavy drinking during mature adulthood (Helms et al., 2014; Baker et al., 2017). Further, the present study extended this finding to an estimate of the dose dependence of the ethanol effect on brain growth in rhesus macaques (Fig. 2).

There are numerous factors that could contribute to the observed impact of chronic alcohol drinking on brain growth. For example, cortical volume, which occupies the greatest volume fraction of the total brain in rhesus macaques (Rakic, 1995; Toro et al., 2008), could account for the observed impact of alcohol on the brain growth. In human cross-sectional data, heavy alcohol drinking was associated with smaller volumes of the lateral frontal and temporal cortices (Squeglia et al., 2015). However, here we established that cortical volume did not increase from 3.9 to 7.5 years in rhesus monkeys (Table 3) and that the global measure of cortical volume was not sensitive to the effects of chronic alcohol drinking. Another significant contributor to the total brain volume is the white matter volume. For example, morphometric studies using MRI have demonstrated that the volume of white matter increases faster with the total brain growth then gray matter volume (Rilling and Insel, 1999). An impact of chronic ethanol on the development of white matter subsequently contributes to the attenuated brain growth. In this study, daily alcohol drinking for over 12 months reduced white matter growth in the NHP brain (Fig. 3), and these results correspond to attenuated growth of white matter in heavy-drinking human subjects (Squeglia et al., 2015). Overall, the unique analyses of growth rate, rather than a single measure of volume, appears to be a key factor in documenting the adverse effects of alcohol on relatively late brain growth studied here.

The volume of subcortical structures also showed age-dependent growth in four of eight subcortical gray matter regions of interest included in the study (specifically, globus pallidus, thalamus, brainstem, and cerebellum). These findings partially correspond to a human cross-sectional pediatric study where the volumes of thalamus, brainstem, and cerebellum increase in a period from 4 to 18 years (Brain Development Cooperative Group, 2012). The data presented here help to establish that subcortical nuclei during adolescence are susceptible to voluntary intake of alcohol. The effect of chronic alcohol drinking on the volumes of subcortical gray matter regions in the human adult brain largely resulted in reduced volumes. Specifically, daily drinking of both heavy and nonheavy amounts of alcohol dramatically reduces the rate of growth in the thalamus, a finding that parallels the effect observed in the adult human brain with reductions in caudate, putamen, thalamus, cerebellum, and hippocampus (Sullivan et al., 2000; Chanraud et al., 2007; Yang et al., 2016). In adolescents, smaller hippocampal, thalamic, and putamen volumes are reported in male alcohol users (Nagel et al., 2005); however, larger thalamic and putamen volumes have been reported in adolescent female drinkers (Fein et al., 2013). In the present study, chronic heavy alcohol drinking attenuated age-related growth of the thalamus in the macaques (Table 5). Overall, the changes in the growth rate of the thalamus and white matter due to chronic alcohol drinking in the macaques have some key similarities to the human data, but the macaque also offers quantitative information related to ethanol dose. Further, the observation that a subset of structures shows an increased rate of volumetric growth suggests that additional studies are needed to understand the allopathic balance between responses of subcortical structures to chronic alcohol use in the late adolescent brain.

In summary, the NHP model of alcohol self-administration, in combination with longitudinal measures by MRI used in this study, highlights the rate of volume changes within the developing primate brain to isolate dose-dependent effects of chronic voluntary alcohol drinking in rhesus macaques. These effects are concentrated within cerebral white matter, and the thalamus is involved in critical control over sensory and limbic integration with behavioral choice and output. Thus, future research is needed to determine whether these volumetric changes might lead to altered functioning within the neural circuitry underlying excessive alcohol drinking.

## Synthesis

Reviewing Editor: Philippe Tobler, University of Zurich Decisions are customarily a result of the Reviewing Editor and the peer reviewers coming together and discussing their recommendations until a consensus is reached. When revisions are invited, a fact-based synthesis statement explaining their decision and outlining what is needed to prepare a revision will be listed below. Note: If this manuscript was transferred from JNeurosci and a decision was made to accept the manuscript without peer review, a brief statement to this effect will instead be what is listed below.

Dear Dr Shnitko, dear Dr. Kroenke,

Thank you for the revision of the JNS version of the article. While the revision was responsive, I noticed a couple of points that should be addressed before going ahead.

1) Please report whether the findings remain the same if you include a regressor of no interest for scanner upgrade

2) ROI-analysis: Please describe the rationale for including the 10 regions as ROI (around line 191) and describe how you accounted for the multiple comparison issue (i.e., correcting for the number of ROIs)
